# How plants manage food reserves at night: quantitative models and open questions

**DOI:** 10.3389/fpls.2015.00204

**Published:** 2015-03-31

**Authors:** Antonio Scialdone, Martin Howard

**Affiliations:** ^1^Wellcome Trust Sanger Institute, Wellcome Trust Genome CampusHinxton, Cambridge, UK; ^2^European Molecular Biology Laboratory-European Bioinformatics Institute (EMBL-EBI), Wellcome Trust Genome CampusHinxton, Cambridge, UK; ^3^Computational and Systems Biology, John Innes CentreNorwich, UK

**Keywords:** *Arabidopsis thaliana*, starch degradation, circadian clock, mathematical modeling, post-translational analog arithmetic

## Abstract

In order to cope with night-time darkness, plants during the day allocate part of their photosynthate for storage, often as starch. This stored reserve is then degraded at night to sustain metabolism and growth. However, night-time starch degradation must be tightly controlled, as over-rapid turnover results in premature depletion of starch before dawn, leading to starvation. Recent experiments in *Arabidopsis* have shown that starch degradation proceeds at a constant rate during the night and is set such that starch reserves are exhausted almost precisely at dawn. Intriguingly, this pattern is robust with the degradation rate being adjusted to compensate for unexpected changes in the time of darkness onset. While a fundamental role for the circadian clock is well-established, the underlying mechanisms controlling starch degradation remain poorly characterized. Here, we discuss recent quantitative models that have been proposed to explain how plants can compute the appropriate starch degradation rate, a process that requires an effective arithmetic division calculation. We review experimental confirmation of the models, and describe aspects that require further investigation. Overall, the process of night-time starch degradation necessitates a fundamental metabolic role for the circadian clock and, more generally, highlights how cells process information in order to optimally manage their resources.

## 1. Introduction

Plants use photosynthesis to assimilate carbon and fuel their metabolism and growth. Atmospheric carbon dioxide is converted into organic compounds, utilizing the energy provided by sunlight. During the night, when photosynthesis is not possible, plants must rely on stored reserves of carbohydrates built up during the previous day. In many plants, this carbohydrate is stored in chloroplastic starch granules which are degraded during the night to produce sugars.

*Arabidopsis thaliana* degrades starch at a constant rate during the night, so that about 95% of all starch is utilized by the time of expected dawn. As many studies have shown (Usadel et al., 2008; Graf et al., 2010; Yazdanbakhsh et al., 2011), this pattern of starch utilization is key for plant productivity: if this process is perturbed by genetic or environmental conditions (such as gene mutations or an artificially prolonged night), the plant shows symptoms of starvation and/or reduced growth.

**Figure 1:**
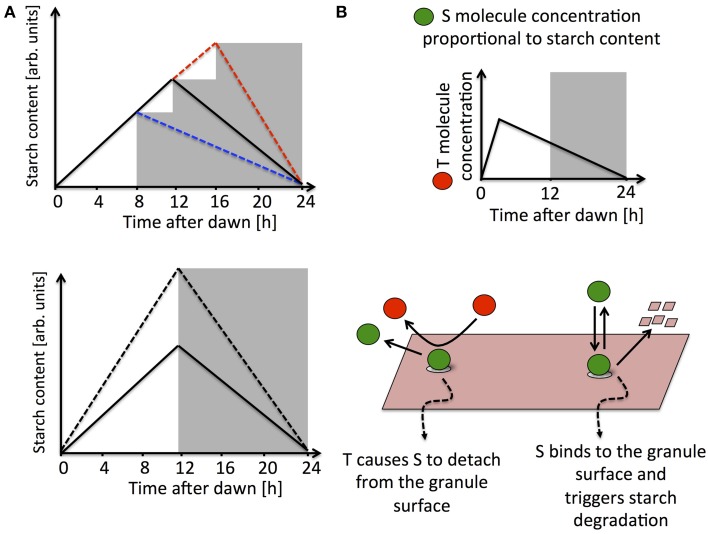
**(A)** Schematic illustrations of the experimental data in Graf et al. (2010) and Scialdone et al. (2013). The upper panel shows how the starch degradation rate is adjusted in an unexpectedly early (blue dashed line) or late (red dashed line) night with respect to a normal 12 h light/12 h dark cycle (solid black line). In the lower panel, starch turnover in a plant exposed to different light intensities is illustrated. The starch degradation rate is immediately recomputed after being exposed to light intensities higher than normal (dashed line compared to full line). **(B)** Simplified representation of a model implementing arithmetic division, for full details see (Scialdone et al., 2013). The *S* molecule concentration is proportional to the starch levels accumulated in the chloroplast, while the *T* molecule, after a resetting-period at the beginning of the day, tracks the time to expected dawn. Starch degradation is promoted by *S*, which binds to the starch granule surface. *T* interacts with *S* causing it to detach from the granule surface, thereby effectively inhibiting the degradation reaction.

The pattern of starch dynamics undergoes long-term changes to allow the plant to adapt to short/long day conditions. Indeed, as the day gets progressively shorter the plant synthesizes starch at a faster rate during the day and degrades it more slowly at night (Gibon et al., 2004). Remarkably, the starch degradation rate is also immediately adjusted in response to many unexpected environmental changes. For instance, if the plant is subjected to an unexpected early or late night, night-time starch degradation proceeds at a slower or faster pace, respectively, to ensure that starch reserves last until the anticipated time of dawn (Graf et al., 2010; Scialdone et al., 2013). Similarly, a change in light intensity that alters the amount of starch produced and available at dusk is immediately compensated by an appropriate change in the rate of night-time degradation (Scialdone et al., 2013, see Figure 1A). The plant circadian clock has an important role in the regulation of starch degradation. Indeed, mutations of circadian clock genes bring about alterations to the normal turnover of starch. For example, the *lhy/cca1* double mutation causes an early depletion of starch reserves at night (Graf et al., 2010).

Despite its importance, the mechanisms that regulate starch degradation remain incompletely understood. The biochemical pathway of starch degradation includes enzymes that catalyze the phosphorylation/dephosphorylation of the starch granule surface and enzymes that hydrolyze the glucose polymers of which the granule is comprised (Stitt and Zeeman, 2012). Notably, while transcripts of these enzymes undergo large diurnal changes, the levels of the encoded proteins do not change significantly over the course of a single day (Smith et al., 2004; Lu et al., 2005; Gibon et al., 2006; Skeffington et al., 2014). Moreover, it has been estimated that it would take days for a change in transcript levels to significantly affect the levels of most of these enzymes (Gibon et al., 2006; Piques et al., 2009; Stitt and Zeeman, 2012). These facts, along with the immediate adjustment of the degradation rate to unexpected perturbations, strongly suggest that the underlying mechanism operates at a post-translational level, possibly through protein modifications, rather than at the level of transcription. In support of this idea, many enzymes involved in starch metabolism show the potential to be modulated by phosphorylation, redox and allosteric regulation (see Kötting et al., 2010; Glaring et al., 2012 and references therein).

Based on these experimental results, mathematical models have recently been developed, analysing the mechanisms that could underlie the computation of the appropriate starch degradation rate and how this operation is controlled by the circadian clock. Many unanswered questions remain, but the models have already produced a number of predictions, some of which have been experimentally verified, and paved the way for the design of future targeted experiments that can potentially identify key regulators of starch degradation. In this review we briefly describe the models, illustrate what they have contributed, and clarify which aspects need further investigation.

## 2. Arithmetic division to prevent starvation

The most striking aspect of the starch degradation process is its ability to immediately compensate for unexpected changes in the length of the night or the amount of starch available to degrade. As recently proposed (Scialdone et al., 2013), such an ability could stem from a mechanism able to keep track of both the expected time to dawn and the starch accumulated, and then to carry out an arithmetic division operation between these two quantities. In this way, the appropriate starch degradation rate can be computed. As discussed above, the mechanism is expected to operate at a post-translational level.

In the simplest scenario, information about starch and time is encoded in two diffusible molecules in the chloroplasts, respectively called *S* and *T*. The experimental evidence suggests the basic features that these two molecules must have. The concentration of molecule *S* should be proportional to the amount of starch present in the chloroplast. This allows a simple and independent measure of the starch accumulated in the granules, much of which is within the granule matrix and is therefore presumably inaccessible to the enzymes involved in starch degradation. The molecule *T* is controlled by the circadian clock and its concentration is proportional to the expected time to dawn Δ*t*, except for a “resetting period” at the beginning of the day (see Figure 1B). *S* and *T* must interact in such a way as to produce a starch degradation rate proportional to the ratio of their concentrations and equal to the ratio between the chloroplastic starch content and the expected time to dawn, i.e., an arithmetic division operation must be implemented. Digital implementation of such an arithmetic operation is prohibitively complex. However, chemical kinetics offers much simpler interaction schemes that can implement arithmetic division in an analog fashion. In one of these interaction schemes, *S* is able to bind to the surface of the starch granule and promote its degradation, possibly through the activation of enzymes, whereas the *T* molecule inhibits degradation by binding to *S* and causing it to detach from the granule surface (Figure 1B). Simple calculations show that under certain conditions that can be explicitly determined, this mechanism leads to a degradation rate proportional to the ratio between the concentrations of *S* and *T* and equal to the amount of chloroplastic starch divided by the expected time to dawn. Moreover, this model can fit very well all the available data (Scialdone et al., 2013).

Other conceptually similar implementations are also possible, and while the details of the mechanism could differ (e.g., depending on how time information is encoded in the dynamics of the *T* molecule), some predictions can still be made. For instance, the model shows that the mechanism is so robust that it can accommodate perturbations even more extreme that those previously tested, such as an unexpected period of light occurring in the middle of the night. This prediction was indeed verified, as it was shown that the starch degradation rate was correctly recomputed after a night-time light period of 5 h (Scialdone et al., 2013). Furthermore, another prediction concerns the properties of starch-excess mutants (i.e., mutants that degrade starch at a slower pace and have a significant amount of starch left over at dawn). Such mutants should still degrade starch linearly with time but consume a fixed percentage of starch during the night, regardless of the starch content at the beginning of the night. This prediction was also experimentally confirmed, with starch degradation mutants like *lsf1* and *sex4* (Comparot-Moss et al., 2010; Smith, 2012) shown to exhibit this phenotype (Scialdone et al., 2013).

## 3. The phosphorylation/dephosphorylation cycle allowing starch degradation

The identity of the hypothesized *S* and *T* molecules is still to be discovered. However, there are experimental clues on where they could be acting. As mentioned above, when a starch granule is degraded, phosphate groups are added by two dikinases, GWD and PWD, and removed by the phosphatases LSF2 and SEX4 (Baunsgaard et al., 2005; Kötting et al., 2005; Ritte et al., 2006; Hejazi et al., 2009; Santelia et al., 2011; Smith, 2012). The addition of phosphate groups is thought to allow access for starch degrading enzymes, with subsequent removal of the phosphate groups needed for full starch polymer degradation. Given that the phosphorylation/dephosphorylation cycle is essential for starch degradation, as it regulates access of enzymes to the granule surface, this cycle is an attractive candidate both for flux modulation and for the storage of information related to starch dynamics. This hypothesis is supported by evidence that the level of phosphate per unit mass of starch is not constant during the day, but rather reaches a maximum around dusk, in dynamics which resembles the behavior predicted for the *S* molecule concentration (Scialdone et al., 2013). In fact, this behavior is also compatible with the dynamics of the *T* molecule concentration in the model outlined above (see Figure 1B). Whether the starch phosphate content mirrors the total amount of starch, or the time to expected dawn, is a question that can be clarified using model predictions. According to the model, in plants that are subjected to an unexpected increase in light intensity, the starch phosphate levels would reach a higher value with respect to the control if the starch phosphate levels were proportional to starch reserves. However, the phosphate levels would be unaltered relative to the control if starch phosphate levels were alternatively proportional to the time to expected dawn.

Another interesting result concerns the phenotype of the mutant lacking PWD. Such a mutant maintains linear starch degradation with time during the night, but is not able to adjust the degradation rate to an unexpectedly early night. This result suggests that PWD could be part of the molecular machinery that sets the degradation rate (Scialdone et al., 2013). Additional experiments will be required to test the behavior of the *pwd* mutant under different conditions (e.g., change in light intensity). The models could then help to interpret these results in order to uncover the exact role of this enzyme. Conversely, GWD has been shown to have little influence over the control of flux in starch degradation, and, in particular, its redox regulation is not necessary for the computation of the appropriate starch degradation rate (Skeffington et al., 2014).

## 4. Investigating the role of the circadian clock

Alternative hypotheses have recently been investigated into how the circadian clock exerts control over starch turnover. The analysis combined models of the *Arabidopsis* circadian clock with the arithmetic division model described above (Seaton et al., 2014).

The first question posed was how information about the time to expected dawn is encoded in the dynamics of the *T* molecule, and, in turn, the role of the *T* molecule in the starch degradation process. One possible behavior is shown in Figure 1B, with the *T* concentration proportional to the time to expected dawn and the *T* molecule acting as an inhibitor of starch degradation. Another possibility is that the *T* concentration increases with time during the day, approximately tracking the time since last dawn. In this case, in order to implement arithmetic division between starch levels and the time to dawn, *T* must promote starch degradation (Scialdone et al., 2013). Possible regulatory schemes capable of producing these *T* dynamics by using circadian clock components are shown in Seaton et al. (2014). Models with a light-gated regulation of starch degradation, where the rate is set at each light-dark transition were also analyzed, as opposed to models with a continuous computation of the starch degradation rate throughout the night. These different scenarios were combined together in model variants, also considering the possibility that starch synthesis might be under the control of the circadian clock. All the model variants explored in Seaton et al. (2014) were capable of reproducing the experimental results so far available. The main differences between them consisted of the phenotypes predicted in the presence of circadian clock mutations. The general conclusion was that models with a light-gated control of starch degradation are more robust to circadian clock mutations than models based on a continuous calculation of the starch degradation rate. On the other hand, if the degradation rate is fixed at dusk, it would not be possible to adjust for fluctuations in reaction rates that could occur at night and cause too rapid or too slow a starch turnover. Robustness to both circadian clock mutations and noise in reaction rates could be combined in a mechanism with an intermediate level of clock control, where the degradation rate is still continuously computed but the *T* molecule is controlled by the clock only during the day and decays linearly with time at night (Seaton et al., 2014).

One possible way to rule out a pure light-gated response, would be to test for the presence of non-linear starch profiles at night in the wild-type. Indeed, a model where the degradation rate is continuously computed predicts that during the resetting-period of the *T* molecule (around dawn, see Figure 1B), linear dynamics cannot take place. Even though some experiments hint at non-linear dynamics, especially around the time of expected dawn (Scialdone et al., 2013), starch measurements with a higher time resolution and precision will be needed to confirm this behavior.

## 5. Coordination between starch degradation and synthesis

Several lines of evidence suggest concerted control of both starch degradation and synthesis. Indeed, plants living in long or short days change both their starch synthesis and degradation rates as compared to plants grown in normal 12 h light/12 h dark cycles. In long days, a comparatively slower starch synthesis rate is associated with more rapid starch degradation, with the opposite pattern occurring in short days (Gibon et al., 2004, 2009; Lu et al., 2005; Sulpice et al., 2014). Moreover, plants that run out of starch after an extended night, synthesize more starch during the next day (Gibon et al., 2004; Mugford et al., 2014).

A recent phenomenological model has investigated this issue, employing sinusoidal cycles governed by the circadian clock for the rates of sucrose export, starch degradation and starch synthesis. Starch turnover adjustments to long/short day conditions could then be reproduced by tuning the relative phases of these cycles in such a way as to minimize sucrose starvation (Feugier and Satake, 2013). Molecular details were not explored, but a degradation rate proportional to the starch content through a time-dependent factor β(t) was imposed. It was noted that a linear with time decrease of starch levels at night could be achieved with a hyperbolic functional form for *β*(*t*) (i.e., *β*(*t*) ~ 1/(*t_day_*−*t*), with *t_day_* being the day length (Feugier and Satake, 2014). This is the form generated by the arithmetic division model through the interaction between the *S* and *T* molecules. Sucrose starvation was also analyzed when one or two among the three rates were constant, where it was shown that regulation of the starch degradation rate was the most important to achieve minimization of sucrose starvation (Feugier and Satake, 2013). Although these conclusions are potentially interesting, the initial assumptions of this model may be too simplistic, as there is little good evidence that the starch synthesis rate is directly under the control of the circadian clock. Indeed, the partitioning of photoassimilate between starch and sucrose is likely to be controlled by multiple mechanisms that may be quite distinct from those ensuring a constant supply of sucrose from starch at night (Mugford et al., 2014; Sulpice et al., 2014). Moreover, it seems unlikely that, as speculated in Feugier and Satake (2013), plants can adjust to different photoperiods through alteration of the phases of clock-dependent metabolic rates, given observations suggesting that there is little change in clock components' phases with photoperiod (Edwards et al., 2010).

A model exploring the molecular mechanisms that could couple starch degradation to other processes in the plant's metabolism has recently been proposed (Pokhilko et al., 2014). This model includes several modules describing metabolism, the circadian clock and interactions between the two. While starch degradation is described by the arithmetic division model, the existence of a molecule *I* is hypothesized, which controls the fraction of photosynthate allocated to starch synthesis. The levels of this molecule *I* are assumed to increase with increasing levels of carbon starvation. In this picture, since regulation of the starch degradation rate imposes a depletion of carbon reserves at the time of expected dawn, an unexpectedly prolonged night would be accompanied by a period of starvation and a consequent increase in the levels of the *I* molecule. This would then induce a higher starch synthesis rate during the following light period, as experimentally observed (see above and Gibon et al., 2004; Mugford et al., 2014). The model predicts the characteristics and behaviors of *I* and of the other presumptive molecular regulators under a number of conditions. Comparisons with experimental data then allowed identification of candidates for some of these molecules (Pokhilko et al., 2014). SnRK1, a kinase that plays an important role in the sucrose synthesis pathway by modulating activity of sucrose-phosphate synthase and F26P phosphatase, was identified as a candidate for *I*. It was also suggested that information about time and carbon status could be integrated into SnRK1 activity through its β−subunit, AKINβ1. This prediction could be validated experimentally by, e.g., inducing overexpression of AKINβ1 which, according to the model, would bring about an increase in the starch synthesis rate (Pokhilko et al., 2014). However, how the levels of these proteins (and not just transcripts) change, and the role of their post-translational modifications, would need to be assessed in order to confirm these putative functions.

## 6. Conclusions

Regulation of carbohydrate availability at night is a crucial aspect of plant metabolism. This is particularly true for annual species like *Arabidopsis*, which complete their life cycle in a year and are under strong selective pressure to optimize carbon utilization for the maximization of seed production. *Arabidopsis* has developed a sophisticated mechanism to control starch turnover during diel cycles which is robust to perturbations in light/dark patterns and also temperature (Pyl et al., 2012). In such a mechanism a central role is played by the circadian clock, and there are suggestions that the regulation of starch degradation is the clock's major contribution to the optimization of plant productivity (Graf and Smith, 2011).

Mathematical modeling is helping to unravel the molecular mechanisms that control starch degradation by generating hypotheses and testable predictions. This approach has been particularly useful in providing a starting point for future experiments for a process whose underlying mechanism was previously completely mysterious. The arithmetic division computation that produces the appropriate starch degradation rate is thought to occur at a post-translational level. Hence it will probably be necessary to analyse the dynamics of the relevant post-translational modifications in order to shed light on the identity of the molecules that implement the computation (Skeffington et al., 2014). Models could then be refined to incorporate additional biochemical details to aid the interpretation and design of new experiments.

However, some model predictions are already supported by experiments, such as the response to previously untested environmental perturbations. Mutants that accumulate abnormally high (e.g., *sex4*) or low (e.g., *pgm*) levels of starch are also important tools that can help to clarify many aspects of starch metabolism (see, e.g., Gibon et al., 2004). In particular, the starch degradation pattern of some starch-excess mutants was used to confirm predictions from the models (see above and Scialdone et al., 2013). Other predictions yet to be tested will help to elucidate important questions, such as whether the computation of the starch degradation rate is a one-off event occurring at dusk or whether it happens continuously during the night, and what is the role for cycles of starch phosphate content. Models have also helped to identify molecules that could have important regulatory functions (e.g., PWD and SnRK1). There have also been efforts to combine models for starch degradation and other aspects of plant metabolism with models at higher scales (e.g., concerning organ growth). The eventual goal here is to construct multiscale models that could provide new insights in plant behavior and help direct plant bioengineering (Chew et al., 2014).

Essentially all plants manufacture and store starch during daylight hours, breaking it down during the night, irrespective of the type of photosynthesis used: C3 (like *Arabidopsis*), C4 or CAM (Weise et al., 2011). Furthermore, evidence suggests that a similarly tight control of starch degradation (linear in time) takes place both in plants having the same type of photosynthesis as *Arabidopsis* (like *Brachypodium distachyon*, see Scialdone et al., 2013), and different types, like *Mesembryanthemum crystallinum*, a facultative CAM plant (Neuhaus and Schulte, 1996). While the biochemical details could differ, models based on the same general concepts could therefore be used to describe starch metabolism in a wide range of cases.

Similar mechanisms that allow optimization of the use of limited resources could also be operating in other biological processes (Scialdone et al., 2013). One example is the glycogen cycle in cyanobacteria (Pattanayak et al., 2014), which exhibits circadian clock-controlled dynamics similar to starch in *Arabidopsis*. Moreover, the use of glycogen reserves is thought to be important for the metabolism of the bacterium at night.

The problem of starch degradation is also a prominent example of how arithmetic computations can be performed in biology in an analog fashion through chemical kinetics (Cory and Perkins, 2008; Buisman et al., 2009). In addition to providing implementations that are simpler than digital alternatives, it has been argued that analog computation is more efficient in terms of energy expenditure and number of molecules required (Sarpeshkar, 2014). The potential of analog dynamics has recently begun to be appreciated in synthetic biology, with the engineering of synthetic analog gene circuits in living cells (Daniel et al., 2013; Sauro and Kim, 2013; Sarpeshkar, 2014). Indeed, the potential importance of analog information processing in these contexts is only now becoming clear and is likely to develop into a subject of intense research interest in the years ahead.

## Author contributions

AS, MH: Drafted the work and revised it critically for important intellectual content.

### Conflict of interest statement

The authors declare that the research was conducted in the absence of any commercial or financial relationships that could be construed as a potential conflict of interest.
